# Longitudinal association of carotid endothelial shear stress with renal function decline in aging adults with normal renal function: A population-based cohort study

**DOI:** 10.1038/s41598-018-38470-x

**Published:** 2019-02-14

**Authors:** Yingxin Zhao, Yuanli Dong, Juan Wang, Lin Sheng, Qiang Chai, Hua Zhang, Zhendong Liu

**Affiliations:** 1 Cardio-Cerebrovascular Control and Research Center, Institute of Basic Medicine, Shandong Academy of Medical Sciences, Jinan, Shandong 250062 China; 2 Department of Community, Lanshan District People Hospital, Linyi, Shandong 276002 China; 3 Department of Cardiology, The Second Hospital of Shandong University, Jinan, Shandong 250000 China

## Abstract

The aim of this study was to investigate the associations between carotid wall shear stress (WSS) and renal function impairment (RFI) and albuminuria in aging adults. A total of 1,447 subjects aged 60 years and older with normal estimated glomerular filtration rate (eGFR ≥ 60 mL·min^−1^·1.72 m^−2^) and albumin/creatinine ratio (ACR < 30 mg·g^−1^) were enrolled between April 2007 and October 2009 in the Shandong area, China. Carotid WSS was assessed at baseline, and eGFR, which is based on serum creatinine and cystatin C, and ACR were assessed at baseline and at the annual follow-up visits. After an average of 62.9 months of follow-up, the reduction in eGFR and the increase in ACR were significantly higher in the Q_1+2+3_ group than the Q_4_ group, as classified by either the interquartile of the mean WSS or the interquartile of the peak WSS after adjustment for multi-variabilities, including the average blood pressures at every annual visit and baseline eGFR and ACR. For groups classified by mean WSS, the hazard ratios (95% confidence intervals) were 3.45 (1.36–8.75, *p* = 0.008) in the incident RFI and 3.24 3.22 (1.37–7.57, *p* = 0.009) in the incident albuminuria for the Q_1+2+3_ group compared with the Q_4_ group. Similar results were observed among groups classified by peak WSS. The Q_1+2+3_ group was associated with endothelial dysfunction and inflammation with respect to the Q_4_ group as classified by mean or peak WSS. The results indicate that carotid WSS plays an important role in RFI and albuminuria progression in aging adults. Lower WSS was associated with a higher risk of RFI and albuminuria compared with higher WSS.

## Introduction

Chronic renal function impairment (RFI) is especially common among older adults^1–4^ and is regarded to be a strong and independent predictor of kidney disease onset^5^. RFI has been defined as an estimated glomerular filtration rate (eGFR) of <60 mL·min^−1^·1.72 m^−2^, along with an albumin/creatinine ratio (ACR) of ≥30 mg·g^−1^^6,7^.

Chronic RFI is thought to share risk factors for atherosclerosis and endothelial dysfunction^4,8,9^. Among these risk factors, an important hemodynamic force called wall shear stress (WSS) plays a crucial role as a fluid mechanical mediator in endothelial cellular activities and vascular remodeling^10^. WSS represents the tangential force per unit area exerted by blood flowing on the endothelial surface of the inner vessel wall in the direction of flow. Studies have demonstrated that WSS regulates the activity of endothelial nitric oxide (NO) synthase and the production of NO, controlling the balance of vasodilator and vasoconstrictor factors^11–13^. Low WSS has been found to contribute to atherogenesis and endothelial dysfunction^11–13^.

Abnormal local hemodynamic environments have been shown to impact the normal mechanotransduction of endothelial cells, triggering the processes of endothelial dysfunction and atherogenesis^14^. Baroreceptors, which are stretch-sensitive fibers located primarily in each of the carotid sinuses near the area where the common carotid artery (CCA) bifurcates, provide a common pathway through which precisely regulated systemic blood flows, and there is hemodynamic change^15,16^. The CCA is a well-established “observation window” for monitoring and measurement of systemic hemodynamic conditions in humans, thereby allowing precise regulation of blood flow^17^, and the carotid WSS may represent the overall hemodynamic condition of renal vessels^9,18,19^. Furthermore, chronic RFI is regarded as a part of a subclinical and generalized atherosclerotic-mediated vascular dysfunction^8,9^.

A previous study indicated that there was a close relationship between carotid WSS and renal function in aging adults^9^. Higher carotid WSS is related to higher eGFR and lower ACR. However, this study was a cross-sectional study and could not clearly illuminate the association between carotid WSS and renal function.

Based on this information, we hypothesized that baseline carotid WSS, serving as a marker of endothelial health, is associated with the development of future kidney disease and albuminuria. The main goal of this study was to conduct a community-based investigation of this association in an aging adult population.

## Results

### Baseline demographic and clinical characteristics

**Figure 1:**
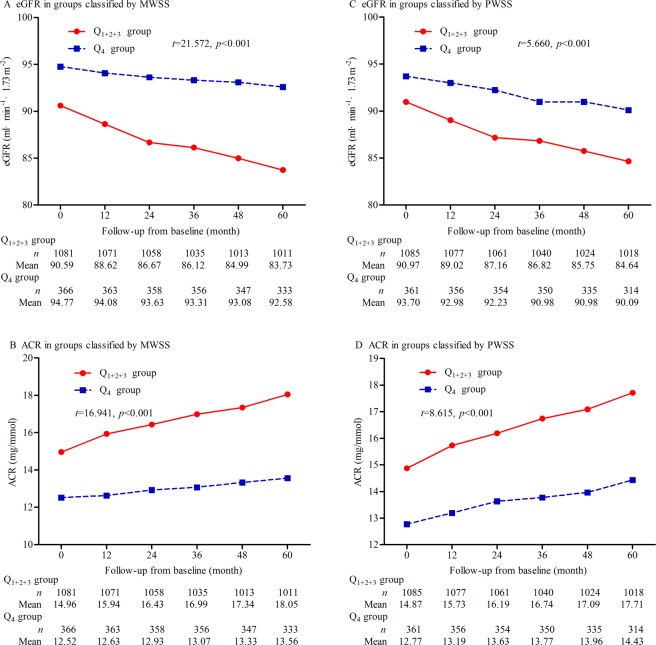
Changes in eGFR and ACR over time. (**A**) Changes in eGFR in the Q_1+2+3_ and Q_4_ groups classified by MWSS, (**B**) changes in ACR in Q_1+2+3_ and Q_4_ groups classified by MWSS, (**C**) changes in eGFR in Q_1+2+3_ and Q_4_ groups classified by PWSS, and (**D**) changes in ACR in Q_1+2+3_ and Q_4_ groups classified by PWSS. eGFR indicates estimated glomerular filtration rate; MWSS, mean wall shear stress; ACR, albumin/creatinine ratio; and PWSS, peak wall shear stress.

Table 1 presents the demographic and clinical characteristics of the participants at baseline. Mean age was 67.92 (SD: 5.56) years; number of females were 724 (50.0%). The average eGFR was 90.56 (IQR: 84.80–98.58) mL·min^−1^·1.73 m^−2^, and the average ACR was 13.85 (IQR: 9.24–19.41) mg·g^−1^. The interquartile of mean WSS was <0.84, 0.84–1.06, 1.07–1.25, and ≥1.26 Pa, and peak WSS was <1.49, 1.49–1.82, 1.83–2.13, and ≥2.14 Pa. Among the 1,447 participants, 1,434 completed at least one follow-up visit during an average of 62.9 (range: 9–66) months of follow-up. eGFR was significantly lower and ACR was higher in the Q_1+2+3_ group than the Q_4_ group, which were classified by MWSS and PWSS separately (all *p* < 0.001, Fig. 1).

**Table 1:** Baseline demographic and clinical characteristics of all participants.

	Grouped by interquartile of MWSS			Grouped by interquartile of PWSS		
	Q_1+2+3_ group (*n* = 1081)	Q4 group (*n* = 366)	*p* value	Q_1+2+3_ group (*n* = 1085)	Q4 group (*n* = 362)	*p* value
Clinical parameters						
Age (years)	68.08 ± 5.72	67.44 ± 5.03	0.055	68.06 ± 5.69	67.49 ± 5.14	0.094
Female [*n* (%)]	543 (50.2)	181 (49.5)	0.797	543 (50.0)	181 (50.0)	0.988
Smoking [*n* (%)]	241 (22.3)	85 (23.2)	0.713	253 (23.3)	73 (20.2)	0.214
Alcohol intake [*n* (%)]	382 (35.3)	153 (41.8)	0.034	392 (36.1)	143 (39.5)	0.288
Hypertension [*n* (%)]	759 (70.2)	183 (50.0)	<0.001	744 (68.6)	198 (54.7)	<0.001
Antihypertensive medication [*n* (%)]	684 (90.1)	168 (91.8)	0.486	673 (90.4)	179 (90.4)	0.982
Diabetes [*n* (%)]	127 (11.7)	44 (12.0)	0.889	123 (11.3)	48 (13.3)	0.326
Lowering-glucose medication [*n* (%)]	120 (94.5)	42 (95.5)	0.805	116 (94.3)	46 (95.8)	0.688
Dyslipidemia [*n* (%)]	286 (26.5)	117 (32.0)	0.042	285 (26.3)	118 (32.6)	0.020
Antidyslipidemia medication [*n* (%)]	140 (49.0)	59 (50.4)	0.788	142 (49.8)	57 (48.3)	0.781
Antiplatelet medication [*n* (%)]	307 (28.4)	92 (25.1)	0.227	297 (27.4)	102 (28.2)	0.767
Body mass index (kg/m^2^)	24.06 ± 2.86	23.89 ± 2.61	0.311	23.96 ± 2.86	24.21 ± 2.61	0.128
SBP (mm Hg)	141.21 ± 13.50	138.11 ± 13.33	<0.001	141.01 ± 13.12	138.58 ± 14.54	0.003
DBP (mm Hg)	70.49 ± 7.07	70.28 ± 7.06	0.619	70.58 ± 7.00	70.01 ± 7.26	0.188
Biochemical parameters						
Total cholesterol (mmol/L)	4.65 ± 0.89	4.65 ± 0.97	0.956	4.62 ± 0.90	4.74 ± 0.93	0.037
Triglycerides (mmol/L)	1.58 ± 0.57	1.63 ± 0.53	0.118	1.59 ± 0.56	1.60 ± 0.54	0.712
HDL-c (mmol/L)	1.14 ± 0.47	1.16 ± 0.51	0.467	1.13 ± 0.47	1.21 ± 0.50	0.003
LDL-c (mmol/L)	2.79 ± 0.72	2.75 ± 0.83	0.360	2.77 ± 0.73	2.80 ± 0.81	0.610
FPG (mmol/L)	5.37 (4.65, 6.22)	5.23 (4.48, 6.06)	0.005	5.29 (4.59, 6.16)	5.44 (4.66, 6.20)	0.056
Creatinine (mg/dL)	0.83 ± 0.19	0.70 ± 0.14	<0.001	0.82 ± 0.19	0.74 ± 0.17	<0.001
Cystatin C (mg/L)	0.97 (0.83, 1.16)	0.79 (0.64, 0.93)	<0.001	0.94 (0.82, 1.14)	0.84 (0.70, 1.01)	<0.001
Urinary albumin (mg/L)	1.52 (0.99, 2.21)	0.79 (0.55, 1.12)	<0.001	1.41 (0.89, 2.08)	0.93 (0.68, 1.46)	<0.001
Endothelial functional and inflammatory parameters						
NO (μmol/L)	50.52 (59.93, 68.49)	76.52 (68.70, 85.52)	<0.001	61.03 (51.05, 70.89)	73.09 (63.07, 81.96)	<0.001
ET-1 (pg/mL)	78.86 (69.29, 87.56)	75.40 (67.58, 84.09)	<0.001	76.99 (67.85, 85.76)	80.05 (72.87, 88.27)	<0.001
ICAM-1 (ng/mL)	137.3 (117.2, 171.0)	134.9 (122.2, 148.8)	0.034	139.1 (119.1, 172.1)	133.2 (117.0, 146.8)	0.001
VCAM-1 (ng/mL)	644.3 (581.9, 732.0)	618.6 (552.4, 688.6)	<0.001	644.3 (575.2, 727.6)	613.8 (556.1, 702.9)	<0.001
hsCRP (mg/L)	3.58 (3.02, 4.20)	2.93 (2.48, 3.57)	<0.001	3.53 (2.93, 4.12)	3.15 (2.65, 3.70)	<0.001
CCA ultrasonographic parameters						
IMT (mm)	1.48 (1.27, 1.72)	1.21 (1.08, 1.37)	<0.001	1.44 (1.22, 1.66)	1.28 (1.13, 1.46)	<0.001
MWSS (Pa)	0.96 (0.80, 1.10)	1.39 (1.32, 1.50)	<0.001	0.98 (0.78, 1.16)	1.30 (1.14, 1.48)	<0.001
PWSS (Pa)	1.67 (1.42, 1.99)	2.21 (1.91, 2.50)	<0.001	1.65 (1.41, 1.90)	2.39 (2.24, 2.54)	<0.001
Renal function parameters						
eGFR (mL·min^−1^·1.73 m^−2^)	89.7 (84.3, 96.7)	95.1 (86.8, 103.0)	<0.001	89.7 (84.4, 97.1)	94.1 (86.4, 102.1)	<0.001
ACR (mg/g)	15.12 (9.86, 20.35)	11.45 (8.91, 15.49)	<0.001	14.82 (9.91, 20.00)	11.76 (8.91, 16.21)	<0.001

Continuous data are described as mean ± standard deviation or median (interquartile range) depending on the normality of the data. Categorical data are described as numbers (percentages). SBP indicates systolic blood pressure; DBP, diastolic blood pressure; HDL-c, high-density lipoprotein cholesterol; LDL-c, low-density lipoprotein cholesterol; FPG, fasting plasma glucose; NO, nitric oxide; ET-1, endothelin-1; ICAM-1, intercellular adhesion molecule-1; VCAM-1, vascular cell adhesion molecule-1; hsCRP, hypersensitive C-reactive protein; CCA, common carotid artery; IMT, intima-media thickness; MWSS, mean wall shear stress; PWSS, peak wall shear stress; eGFR, estimated glomerular filtration rate based on creatinine and cystatin C; and ACR, albumin/creatinine ratio.

### Changes in eGFR and ACR over time

In the Q_1+2+3_ and Q_4_ groups classified by MWSS, eGFR decreased and ACR increased at follow-up compared to the baseline. The reduction in eGFR and the increase in ACR were significantly higher in the Q_1+2+3_ group than the Q_4_ group after adjustment for age; sex; smoking; alcohol intake; histories of hypertension, diabetes, and dyslipidemia; medications of antihypertension, anti-dyslipidemia, glucose-lowering, and antiplatelet; and baseline blood pressures, blood lipids, fasting plasma glucose, and CCA-IMT (all adjustment *p* < 0.001, Fig. 1). However, the participants were grouped by PWSS, the reduction in eGFR and the increase in ACR were higher in the Q_1+2+3_ group than the Q_4_ group after adjustment of multi-variabilities (all adjustment *p* < 0.001, Fig. 1).

### Study outcomes over time

**Figure 2:**
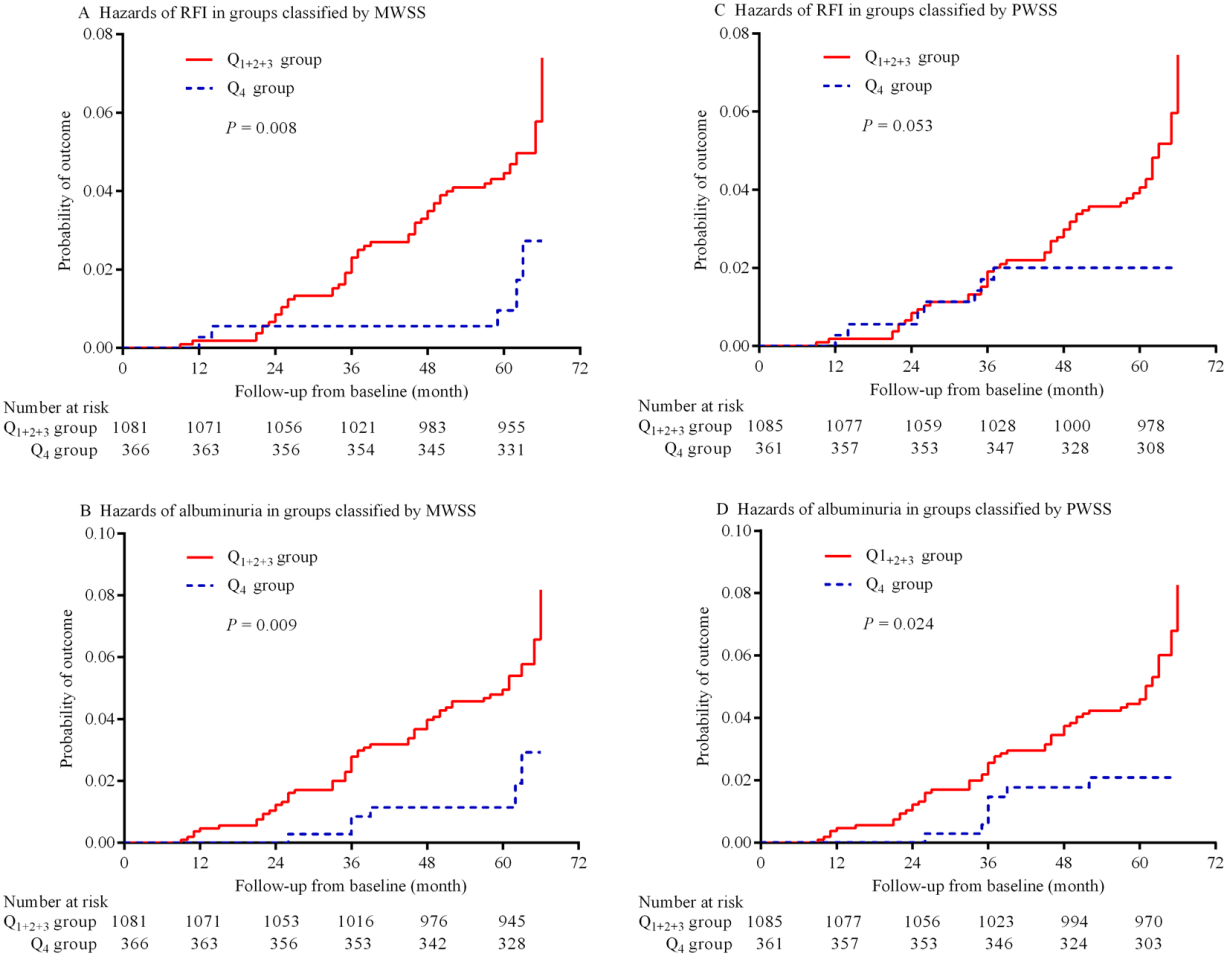
Probability hazards of RFI and albuminuria over time. (**A**) Probability of the incidence of RFI in the Q_1+2+3_ and Q_4_ groups classified by MWSS, (**B**) probability of the incidence of albuminuria in the Q_1+2+3_ and Q_4_ groups classified by MWSS, (**C**) probability of the incidence of RFI in the Q_1+2+3_ and Q_4_ groups classified by PWSS, and (**D**) probability of the incidence of albuminuria in the Q_1+2+3_ and Q_4_ groups classified by PWSS. RFI indicates renal function impairment; MWSS, mean wall shear stress; and PWSS, peak wall shear stress.

Of the 1,434 participants, 54 (3.8%) developed RFI and 61 (4.3%) developed albuminuria over the follow-up period. The incidences of RFI and albuminuria were significantly higher in the Q_1+2+3_ group than in the Q_4_ group, which were grouped by MWSS after adjustment for multi-variabilities including baseline eGRF and ACR and the average blood pressures at every annual visit [for RFI: 49 (4.6%) *vs*. 5 (1.4%), *p* = 0.005 and for albuminuria: 55 (5.1%) *vs*. 6 (1.7%), *p* = 0.007]. The cumulative HRs (95% CIs) were 3.45 (1.36–8.75, *p* = 0.008) in the incidence of RFI and 3.22 (1.37–7.57, *p* = 0.009) in the incidence of albuminuria for the Q_1+2+3_ group compared with the Q_4_ group after adjustment for multi-variabilities (Fig. 2). While the participants were grouped by PWSS, the incidences of RFI and albuminuria were 47 (4.4%) and 54 (5.0%) in the Q_1+2+3_ group versus 7 (2.0%) and 7 (2.0%) in the Q_4_ group (*p* = 0.048 and 0.017, respectively). The cumulative HRs (95% CIs) were 2.16 (0.99–4.71, *p* = 0.053) in the incidence of RFI and 2.49 (1.13–5.49, *p* = 0.024) in the incidence of albuminuria for the Q_1+2+3_ group compared with the Q_4_ group after adjustment for multi-variabilities (Fig. 2).

To investigate the effect of hypertension, dyslipidemia, and diabetes on the association between CCA WSS and the incidences of RFI and albuminuria, we stratified the participants in models (Table 2). The cumulative HRs in the incidences of RFI and albuminuria were significantly higher in the Q_1+2+3_ group compared with the Q_4_ group as classified by either MWSS or PWSS (all *p* < 0.05), except for the cumulative HRs in the incidences of RFI between the Q_1+2+3_ and Q_4_ groups of the participants without dyslipidemia and diabetes as classified by PWSS (*p* = 0.083 and 0.053, respectively) after adjustment for multi-variabilities.

**Table 2:** Probability hazards of RFI and albuminuria over time stratified by hypertension, dyslipidemia, and diabetes.

	Grouped by MWSS				Grouped by PWSS			
	Incidence of RFI		Incidence of albuminuria		Incidence of RFI		Incidence of albuminuria	
	HR (95% CI)	*P* value	HR (95% CI)	*P* value	HR (95% CI)	*P* value	HR (95% CI)	*P* value
**Stratified by hypertension**								
Not hypertension								
Q_1+2+3_ group *vs*. Q_4_ group	1.758 (1.006, 3.072)	0.038	5.045 (1.165, 21.839)	0.030	1.238 (1.001, 1.531)	0.041	1.303 (1.009, 1.683)	0.027
Hypertension								
Q_1+2+3_ group *vs*. Q_4_ group	4.979 (1.203, 20.603)	0.027	2.365 (1.044, 5.358)	0.011	3.485 (1.077, 11.277)	0.037	5.376 (1.299, 22.247)	0.020
**Stratified by dyslipidemia**								
Not dyslipidemia								
Q_1+2+3_ group *vs*. Q_4_ group	3.641 (1.118, 11.855)	0.032	3.960 (1.221, 12.842)	0.022	1.553 (0.948, 2.544)	0.083	2.116 (1.029, 4.351)	0.017
Dyslipidemia								
Q_1+2+3_ group *vs*. Q_4_ group	3.275 (1.349, 7.951)	0.015	2.647 (1.280, 5.474)	0.019	3.535 (1.002, 12.472)	0.047	3.880 (1.001, 15.039)	0.048
**Stratified by diabetes**								
Not diabetes								
Q_1+2+3_ group *vs*. Q_4_ group	4.869 (1.507, 15.725)	0.008	2.658 (1.134, 6.230)	0.025	2.510 (0.989, 6.369)	0.053	2.417 (1.031, 5.665)	0.042
Diabetes								
Q_1+2+3_ group *vs*. Q_4_ group	2.405 (1.008, 5.738)	0.044	1.911 (1.071, 3.410)	0.037	1.524 (1.000, 2.323)	0.49	3.369 (1.026, 11.063)	0.025

RFI indicates renal function impairment; MWSS, mean wall shear stress; and PWSS, peak wall shear stress.

The models were adjusted for age; sex; smoking; alcohol intake; medications for antihypertension, anti-dyslipidemia, glucose-lowering, and antiplatelet; baseline blood pressures, blood lipids, fasting plasma glucose, common carotid artery intima-media thickness, estimated glomerular filtration rate, and albumin/creatinine ratio; and the average systolic and diastolic blood pressure levels at every annual visit.

### Changes in endothelial function and chronic inflammation over time

**Figure 3:**
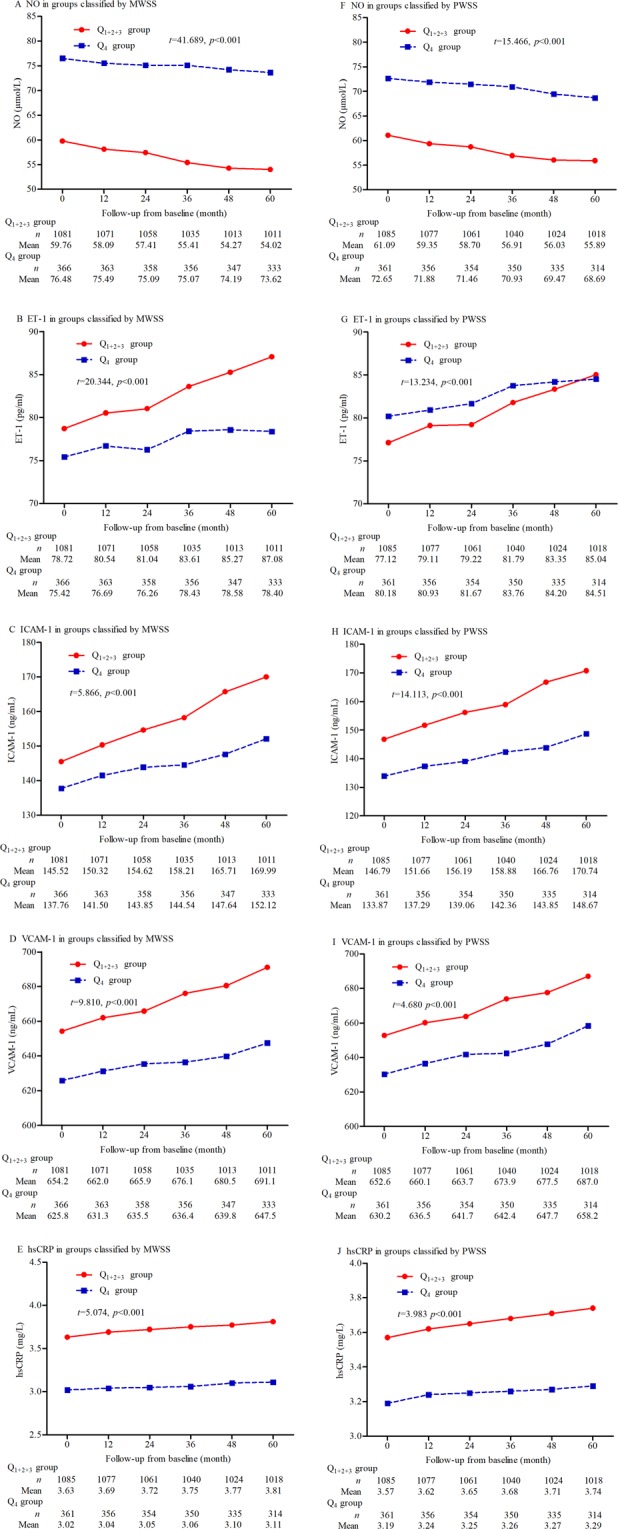
Changes in endothelial function and chronic inflammation over time. (**A**) Changes in NO in the Q_1+2+3_ and Q_4_ groups classified by MWSS, (**B**) changes in ET-1 in the Q_1+2+3_ and Q_4_ groups classified by MWSS, (**C**) changes in ICAM-1 in the Q_1+2+3_ and Q_4_ groups classified by MWSS, (**D**) changes in VCAM-1 in the Q_1+2+3_ and Q_4_ groups classified by MWSS, (**E**) changes in hsCRP in the Q_1+2+3_ and Q_4_ groups classified by MWSS, (**F**) changes in NO in the Q_1+2+3_ and Q_4_ groups classified by PWSS, (**G**) changes in ET-1 in the Q_1+2+3_ and Q_4_ groups classified by PWSS, (**H**) changes in ICAM-1 in the Q_1+2+3_ and Q_4_ groups classified by PWSS, (**I**) changes in VCAM-1 in the Q_1+2+3_ and Q_4_ groups classified by PWSS, and (**J**) changes in hsCRP in the Q_1+2+3_ and Q_4_ groups classified by PWSS. MWSS indicates mean wall shear stress; PWSS, peak wall shear stress; NO, nitric oxide; ET-1, endothelin-1; ICAM-1, intercellular adhesion molecule-1; VCAM-1, vascular cell adhesion molecule-1; and hsCRP, hypersensitive C-reactive protein.

In this study, we also assessed the differences in the changes in endothelial function and chronic inflammation over time between groups classified by MWSS and PWSS separately (Fig. 3). In the Q_1+2+3_ and Q_4_ groups, which were classified by MWSS and PWSS separately, serum NO levels decreased and serum ET-1, ICAM-1, VCAM-1, and hsCRP levels over the follow-up period significantly increased in the Q_1+2+3_ group compared with the Q_4_ group (all adjustment *p* < 0.001).

### Changes in blood pressures, lipids, and fasting plasma glucose over time

**Figure 4:**
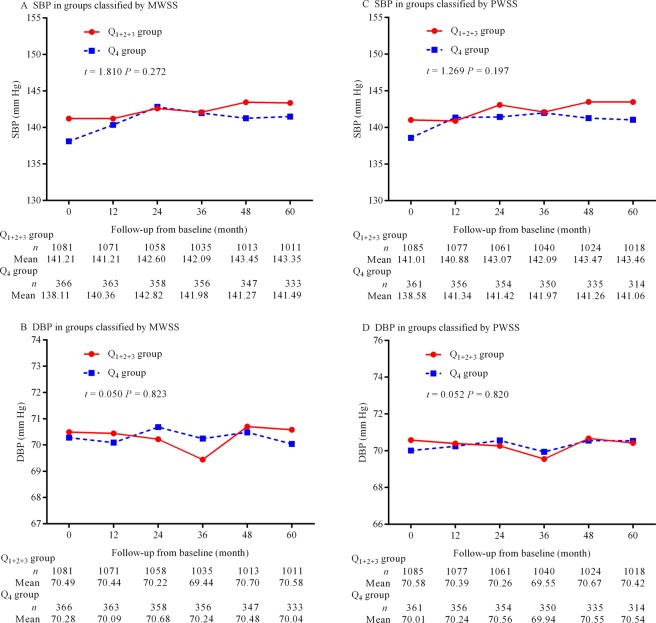
Changes in blood pressure over time. (**A**) Changes in SBP in the Q_1+2+3_ and Q_4_ groups classified by MWSS, (**B**) changes in DBP in the Q_1+2+3_ and Q_4_ groups classified by MWSS, (**C**) changes in SBP in the Q_1+2+3_ and Q_4_ groups classified by PWSS, and (**D**) changes in DBP in the Q_1+2+3_ and Q_4_ groups classified by PWSS. MWSS is mean wall shear stress. PWSS indicates peak wall shear stress; SBP, systolic blood pressure; and DBP, diastolic blood pressure.

**Figure 5:**
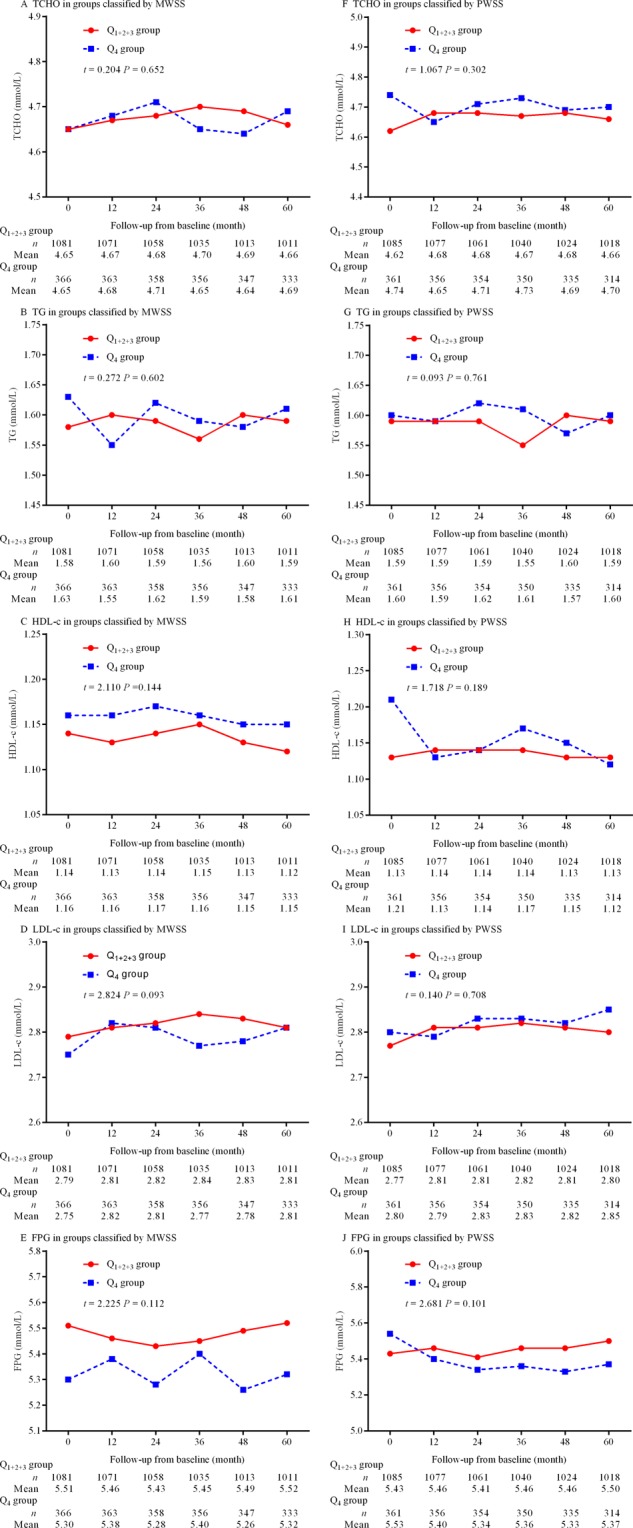
Changes in plasma levels of lipids and glucose over time. (**A**) Changes in TCHO in the Q_1+2+3_ and Q_4_ groups classified by MWSS, (**B**) changes in TG in the Q_1+2+3_ and Q_4_ groups classified by MWSS, (**C**) changes in HDL-c in the Q_1+2+3_ and Q_4_ groups classified by MWSS, (**D**) changes in LDL-c in the Q_1+2+3_ and Q_4_ groups classified by MWSS, (**E**) changes in FPG in the Q_1+2+3_ and Q_4_ groups classified by MWSS, (**F**) changes in TCHO in the Q_1+2+3_ and Q_4_ groups classified by PWSS, (**G**) changes in TG in the Q_1+2+3_ and Q_4_ groups classified by PWSS, (**H**) the changes in HDL-c in the Q_1+2+3_ and Q_4_ groups classified by PWSS, (**I**) changes in LDL-c in the Q_1+2+3_ and Q_4_ groups classified by PWSS, and (**J**) changes in FPG in the Q_1+2+3_ and Q_4_ groups classified by PWSS. MWSS indicates mean wall shear stress; PWSS, peak wall shear stress; TCHO, total cholesterol; TG, triglycerides; HDL-c, high-density lipoprotein cholesterol; LDL-c, low-density lipoprotein cholesterol; and FPG, fasting plasma glucose.

Figure 4 shows the differences in the changes in blood pressure, and Fig. 5 depicts the differences in the changes in lipids and fasting plasma glucose over time. No significant differences between groups classified by MWSS and PWSS were observed (all *p* > 0.05).

## Discussion

In this study, the major findings were that the lower mean and peak WSSs of CAA at baseline were significantly associated with (1) the subsequent reduction in eGFR and increase in ACR, (2) higher risks of the incidences of RFI and albuminuria, even after adjustment for confounders, and (3) the progression of vascular endothelial dysfunction and chronic low-grade inflammation.

In recent decades, the association between the local rheologic force of circulating blood and chronic kidney diseases has received increasing attention. Park^19^ and Verbeke^20^ reported that the mean and maximum wall shear rates of the brachial artery in healthy subjects were significantly higher than those in subjects with chronic kidney disease and/or end-stage renal disease, even though their blood flow rates were similar. In the CCA, WSS was markedly lower in the prior-to-hemodialysis patients with end-stage renal failure than in the presumed healthy age- and sex-matched control subjects^21^. Our previous study showed that lower carotid artery WSS was closely correlated with lower eGFR and higher ACR^9^. The above-mentioned studies suggested that there is a close relationship between local WSS and chronic kidney diseases. However, it was difficult to illuminate the association between the local WSS and chronic kidney diseases in these cross-sectional studies.

In this study, we found that eGFR significantly decreased and ACR significantly increased over time with lower WSS compared with higher WSS, as classified by either MWSS or PWSS. After an average of 62.9 months of follow-up, the incidences of RFI and albuminuria were more than three-fold in the Q_1+2+3_ MWSS group than the Q_4_ MWSS group and more than two-fold in the Q_1+2+3_ PWSS group compared with the Q_4_ PWSS group, even after adjustment for confounders, including baseline CCA-IMT, eGFR, and ACR and the average blood pressure at each annual follow-up visit. Although there were no significant differences in the incidences of RFI in participants without dyslipidemia and diabetes between the Q_1+2+3_ and Q_4_ groups, the risks of RFI and albuminuria were higher in the Q_1+2+3_ group than the Q_4_ group as classified by MWSS and PWSS in participants with/without hypertension, with dyslipidemia, and with diabetes. Moreover, consistent with our previous study^9^, eGFR was significantly lower and ACR was higher in the Q_1+2+3_ groups than in the Q_4_ groups as classified by MWSS and PWSS, respectively. The results indicated that carotid WSS might be important for the development and progression of RFI and albuminuria in older populations.

Chronic kidney disease and/or end-stage renal disease negatively affects vascular function and thus causes divergent vascular pathologies, including atherosclerosis, endothelial dysfunction, vascular calcification, and hemodynamic disturbance^19–21^. However, whether hemodynamic disturbances such as disturbances in carotid WSS affect the decline of renal function in subjects with normal renal function remain unclear. In this study, all participants who were eligible and enrolled at baseline had eGFR ≥ 60 mL·min^−1^·1.72 m^−2^ and ACR < 30 mg·g^−1^. These results suggested that carotid artery rheologic forces might play a crucial role in the development of RFI and albuminuria in aging adults with normal renal function. Among the many reasons for the association between the disturbances in carotid WSS and RFI, the primary reason might be that lower WSS upregulates endothelial dysfunction and inflammation that represents a marker of vascular damage^9,11–13,22^.

Hemodynamic forces are essential for vascular endothelial functions. Associations have been well established between lower and/or oscillatory shear stress and endothelial dysfunction^23^. In this study, changes in endothelial function and chronic inflammation over time were assessed using serum biomarkers. The results showed that serum NO levels decreased, and serum ET-1, ICAM-1, VCAM-1, and hsCRP levels significantly increased in the Q_1+2+3_ group compared with the Q_4_ group that were classified either by MWSS or by PWSS. ACR is not only one of the renal functional markers but is also regarded as one of the most important markers of generalized endothelial dysfunction^24,25^. Endothelial dysfunction leads to impairment of the endothelial barrier, which results in increased transvascular albumin leakage^24,26^.

Endothelial dysfunction has been regarded as an early event in atherosclerosis and is associated with kidney disease^27–29^. Endothelial dysfunction results in the production of reactive oxygen species; increased endothelial permeability; increased intracellular Ca^2+^ concentrations; and expression of cytokines, chemokines, and adhesion molecules, which facilitate leukocyte transmigration into the extracellular matrix underneath, and finally vascular calcification and RFI^27,30–32^.

We did not find the significant differences in the changes in blood pressures and plasma levels of lipids and glucose between groups classified by MWSS and PWSS in the duration of follow-up in this study. This may be attributable to the fact that this study was an observational study, and we were unable to control for medication schedules such as treatment for hypertension, dyslipidemia, or diabetes. However, our results further demonstrated that endothelial shear stress could play an important role in the progression of renal function decline.

A major strength of this study is that it is a longitudinal prospective cohort study. It enabled us to explore the potential longitudinal associations between WSS and RFI progression in subjects with normal renal function that are difficult to explain by cross-sectional relationships. Another is that we have combined serum creatinine and cystatin C to estimate GFR. The precision of these equations has been validated within the Chinese population and can be used to reduce the bias that results from GFR estimated by creatinine and cystatin C alone^33–35^.

This study has several limitations. First, the participants were predominantly recruited from the Han ethnic population in the Shandong area, China. There were essentially ethnic, racial, and geographical limitations. Genetic background has been reported to be strongly associated with eGFR^36^. Thus, multi-ethnic and multi-national studies are needed. Second, we did not measure the renal arteries’ WSS. The renal arteries’ WSS tends to be more reasonable, which could be a precise reflection of the renal rheological forces in comparison to the carotid WSS. Third, RFI is a chronic disease with multi-risk factors^37,38^. Thus, the histories and medications of hypertension, diabetes, and dyslipidemia and the antiplatelet medication were accounted for in the analysis models, as these diseases and medications might influence the carotid WSS and renal function and introduce a certain bias^39^. In addition, we did not investigate the exercise and dietary habits of patients, which may affect the carotid WSS and renal function^40^.

In conclusion, our findings suggested that CCA WSS plays an important role in RFI progression in aging adults with normal renal function. Lower WSS was associated with a higher risk of RFI compared with higher WSS. This association might be via vascular endothelial dysfunction and low-grade inflammation pathways.

## Patients and Methods

### Study population and design

This study was based on data from an observational and population-based cohort study in the Shandong area, China^41^. The major objectives of the original cohort were to investigate determinants of various chronic diseases in a general population with ages 15 years and older (Registration number: ChiCTR-EOC-17013598), and the cohort was expanded to 21,000 participants between April 2007 and October 2009. The exclusion criteria of this study were as follows: age < 60 years, eGFR < 60 mL·min^−1^·1.72 m^−2^ and ACR ≥ 30 mg·g^−1^, secondary hypertension, heart failure, active malignancy, severe liver disease, including chronic hepatitis and cirrhosis, and drug abuse. Finally, 1,447 older subjects were eligible and enrolled in this study. This study was approved by the Research Ethics Committee of the Institute of Basic Medicine, Shandong Academy of Medical Sciences and compliance with the “Declaration of Helsinki”. Written informed consents were obtained from all participants.

### Ultrasonography of common carotid artery and WSS evaluation

Ultrasound examinations were performed by experienced ultrasonographers who were blinded to the participants’ clinical details and during morning hours in a quiet room with a temperature between 22 °C and 25 °C. Before examination, the participants were not allowed to use/consume vasoactive medications (including nitrates, calcium antagonists, angiotensin-converting enzyme inhibitors, and angiotensin antagonists), smoke, alcohol, tea, and caffeine for 24 h and underwent fasting for 12 h. Duplex ultrasonography of the left and right common carotid artery was examined using a high-resolution ultrasound with a 7.5-MHz linear array transducer and electrocardiogram triggering (Vivid *i*, GE Medical Systems Ultrasound Israel Ltd., Tirat Carmel, Israel). Intima-media thickness of CCA (CCA-IMT) was measured by recording ultrasonographic images on the far wall of the CCA. CCA-IMT was defined as the distance from the interface of the CCA lumen-intima (first echogenic line) to the collagen-containing upper layer of the CCA adventitia (second echogenic line). Peak systolic velocity (V_PS_), end diastolic velocity (V_ED_), and mean velocity (V_M_) were measured 1–2 cm below the bifurcation for three cardiac cycles, and their computed means were used for further analysis. The internal diameter (ID) was defined as the distance from the leading edge of the echo produced by the intima-lumen interface of the near wall to the leading edge of the echo produced by the lumen-intima interface of the far wall^9,18,42^. The IDs at the R (ID_R_) and peak T (ID_T_) waves on the electrocardiogram were acquired using B-mode tracings with automatic border detection function. The carotid artery wall was assumed to be rigid with blood as a Newtonian fluid^42^, and WSS was calculated using the following formula^9,43,44^: mean WSS = (8 × η × V_M_/ID_R)_ and peak WSS = (8 × η × V_PS_/ID_T_), where WSS is the carotid artery wall shear stress (Pa), η is the blood viscosity and equal to 0.0035 Pa·s; V is the velocity (m/s), and ID is the lumen diameter (m). WSS was calculated separately for mean and peak systolic velocity.

### Estimated glomerular filtration rate evaluation

Serum creatinine and cystatin C were assessed annually using an enzymatic method (Shanghai Kehua Dongling Diagnostic Products Co., Ltd., China) and the particle-enhanced immunoturbidimetry assay (Beijing Leadman Biomedical Co., Ltd., China), respectively. In this study, we used a combination of serum creatinine and cystatin C to estimate the GFR using the Chronic Kidney Disease Epidemiology Collaboration equation: eGFR (mL·min^−1^·1.73 m^−2^) = 135 × min(creatinine/κ, 1)^α^ × max(creatinine/κ, 1)^−0.601^ × min(cystatin C/0.8, 1)^−0.375^ × max(cystatin C/0.8, 1)^−0.711^ × 0.995^Age^ [×0.969 if female] [×1.08 if black], where κ is 0.7 for females and 0.9 for males, α is −0.248 for females and −0.207 for males, min indicates the minimum of creatinine/κ or 1, and max indicates the maximum of creatinine/κ or 1^33^.

### Urinary albumin excretion assessment

Urinary albumin excretion was assessed annually using urinary ACR that was calculated using the urinary albumin from the morning first void sterile spot and serum creatinine as previously described^9^. The threshold of albuminuria was 30 mg·g^−1^ of ACR in categorical analyses.

### Vascular endothelial function and chronic inflammation assessment

In this study, the vascular endothelial function and chronic inflammation were assessed using the serum levels of NO, endothelin (ET)-1, intercellular adhesion molecule (ICAM)-1, vascular cell adhesion molecule (VCAM)-1, and hypersensitive C-reactive protein (hsCRP). NO levels were assessed using the quantification of nitrite by Griess assay^45^. The reagents were purchased from Sigma (St. Louis, MO, USA). ET-1, ICAM-1, VCAM-1, and hsCRP levels were assessed using enzyme-linked immunosorbent assay kits (Bender MedSystems, Vienna, Austria) following the manufacturer’s instructions. Each sample was tested in duplicate, and the mean value was used for further analysis.

### Follow-up

With the help of family physicians and nurses, participants were visited annually after the baseline survey. Demographic and clinical characteristics of participants including medications (antihypertensive, anti-dyslipidemia, glucose-lowering, and antiplatelet) were acquired at baseline and every annual follow-up visit. For the participant with hypertension, dyslipidemia, or diabetes, the corresponding therapy had been advised at every follow-up visit when the participant was willing to receive treatment. However, there was no unified therapeutic regimen in our study.

### Outcomes

The progression of renal dysfunction and albuminuria was assessed using the changes in eGFRs and ACR, respectively, over time. Putative RFI was identified as eGFR < 60 mL·min^−1^·1.72 m^−2 ^^7^. Putative albuminuria was identified as ACR ≥ 30 mg·g^−1^ ^6,7^.

### Statistical analysis

SPSS for Windows software package, version 24.0 (SPSS Inc., Chicago, IL, USA), was used to perform the statistical analyses. The continuous data were expressed as means and standard deviations or median with interquartile range (IQR, range: 25th to 75th percentile) depending on the normality of the data, which was determined by the Kolmogorov-Smirnov test. Categorical data were presented as numbers with percentages. Participants were classified into two groups (Q_4_ group *vs*. Q_1+2+3_ group) by the interquartile of mean WSS and peak WSS separately. The comparisons of continuous data between the groups were performed using the Student’s *t*-test or Wilcoxon W test depending on the normality of the data. The chi-square test was used to assess the differences in categorical data. A linear mixed model was used to compare the changes in eGFR, ACR, and endothelial function over time among the groups. A logistic regression model was used to compare the odds of the incidences of RFI and albuminuria among the groups. Cumulative incidences of RFI and albuminuria were assessed using the Kaplan-Meier method and comparisons were assessed using log-rank test among groups. The hazard ratios (HRs) with 95% confidence interval (CI) in the incidences of RFI and albuminuria were evaluated using Cox proportional hazards models after adjustment for age; sex (male *vs*. female); smoking (no *vs*. yes); alcohol intake (no *vs*. yes); histories of hypertension, diabetes, and dyslipidemia (no *vs*. yes); medications for antihypertension, anti-dyslipidemia, glucose-lowering, and antiplatelet (no *vs*. yes); baseline blood pressures, blood lipids, fasting plasma glucose, CCA-IMT, eGFR, and ACR; and the average blood pressures at every annual visit. A two-sided *p* < 0.05 was considered statistically significant.
